# Potential drug–drug interactions among elderly patients admitted to medical ward of Ayder Referral Hospital, Northern Ethiopia: a cross sectional study

**DOI:** 10.1186/s13104-016-2238-5

**Published:** 2016-09-01

**Authors:** Fantaye Teka, Gebrehiwot Teklay, Eskindeir Ayalew, Terefe Teshome

**Affiliations:** 1 Forecasting and Capacity Building Directorate, The Federal Democratic Republic of Ethiopia Pharmaceutical Fund and Supply Agency, Addis Ababa, Ethiopia; 2 Clinical Pharmacy Course and Research Unit, Department of Pharmacy, College of Health Sciences, Mekelle University, Mekelle, Ethiopia; 3 Pharmacoepidemiology and Social Pharmacy Course and Research Unit, Department of Pharmacy, College of Health Sciences, Mekelle University, Mekelle, Ethiopia

**Keywords:** Elderly, Drug–drug interaction, Polypharmacy, Ethiopia

## Abstract

**Background:**

The elderly are considered as special population, as they differ from younger adults in terms of comorbidity, polypharmacy, pharmacokinetics, vulnerability to drug–drug interactions and adverse drug reactions. Despite the fact that the elderly patients are at high risk of having drug interaction and potential adverse outcomes, studies in this regard are scarce in resource limited settings like Ethiopia. The aim of this study was to assess the prevalence and determinants of potential drug–drug interaction in elderly patients admitted to medical ward of Ayder Referral Hospital in Northern Ethiopia.

**Methods:**

A cross sectional study was conducted among elderly inpatients aged 60 years and above. The study was conducted from February to May 2014. Prescribed drugs being taken concurrently for at least 24 h were included and checked for drug–drug interaction using Micromedex® 2.0 online drug reference. Data were analyzed using statistical software, statistical package for social sciences for windows version 20. Logistic regression model was used to analyze factors associated with occurrence of drug interaction. P value of <0.05 was considered statistically significant.

**Results:**

A total of 140 patients were participated in the study. The mean age (±standard deviation) of participants was 68 (±7) years. Majority (61.4 %) of patients were diagnosed with cardiovascular and/or renal diseases. A total of 814 drugs were prescribed with a mean of 6 (±4) medications per patient during a 13 (±9) days of hospital stay. About two-third (62.2 %) of the respondents were exposed to at least one potential drug–drug interaction. Among these 3.6, 32.9 and 25.7 % of patients had taken contraindicated drug combination, at least one major and at least one moderate drug–drug interaction, respectively. Patients with five or more prescribed medications were four times at risk of having drug–drug interaction (P = 0.00; adjusted odds ratio 4.047; 95 % confidence interval 1.867–8.775).

**Conclusion:**

Drug–drug interaction in elderly patients was common in this resource limited set-up. Awareness creation and clinical pharmacist involvement in minimizing the risk associated with potentially harmful drug combinations are needed.

**Electronic supplementary material:**

The online version of this article (doi:10.1186/s13104-016-2238-5) contains supplementary material, which is available to authorized users.

## Background

The ageing process is biological reality that has its own dynamic, largely beyond human control. The age of 65 and above, roughly equivalent to retirement ages in most developed countries is said to be the beginning of old age. However, this is not adapted well to the situation in developing countries like Africa. The United Nation (UN) agreed cutoff is 60 and above to refer to the older population [1] and this definition has gained acceptance in Ethiopian context as it coincides with the country’s official retirement age [2].

The elderly differ from young adults in terms of comorbidity, polypharmacy, pharmacokinetics and greater vulnerability to adverse drug reactions (ADRs) [3]. Clinical trials are usually done in young adults excluding the elderly population. This brings a challenge in caring the elderly as findings obtained from trials may not be applicable to all real patients [4]. Along with age related gradual changes, the pharmacodynamics and pharmacokinetics of drugs in the elderly is altered. There are physiological changes that affect how medicines are handled, including alterations in drugs absorption, volumes of distribution, metabolism and clearance which can prolong half-life, increase potential for drug toxicity and the likelihood of ADRs [5–7]. In addition, there is alteration in drug response or sensitivity in the elderly probably due to changes in receptor numbers, changes in receptor affinity and age-related impairment of homeostatic mechanisms [4, 8].

Drug–drug interactions (DDIs) occurs when the effects of one drug is altered by another concomitantly administered drug [9]. The effect of a DDI might be apparent as decline in therapeutic effect of a drug, increased occurrence of ADRs and compromised treatment outcomes [10, 11]. Advanced age, polypharmacy and multiple prescribers have been identified as risk factors for occurrence of potential drug interactions [12]. DDIs are more likely to happen in the elderly because they tend to use multiple medications and have altered pharmacokinetics [6, 13]. A systematic literature review by Saedder et al. also identified number of drugs as the most frequently documented independent patient-related risk factor for serious ADRs in both the general adult population as well as in the elderly [14].

Drug interactions are considerable cause of ADRs and hospital admission. The incidence of DDI related ADRs in the elderly had been estimated to ranges from 4.5 to 6.5 % [15, 16]. In a systematic review and meta-analysis of observational study, DDI accounted for the 1.1 % hospital admissions and 0.1 % hospital visits [17]. More than half of all hospitalizations due to ADR had occurred in the elderly patients [18, 19]. Furthermore, DDI is associated with increased length of hospital stay and other added healthcare costs [20–22]. Despite the fact that the elderly patients are at high risk of having drug interaction and potential adverse outcomes, studies in this regard are scarce in resource limited settings like Ethiopia. The aim of this study was to assess the prevalence and determinants of potential DDIs in elderly patients admitted to medical ward of Ayder Referral Hospital (ARH) in Northern Ethiopia.

## Methods

The study was conducted from February to May 2014 in ARH located in Mekelle city of Tigray Regional State, Northern Ethiopia. ARH is a teaching and the highest level medical facility in the northern part of the country. It provides general inpatient and outpatient services in four major departments (Internal Medicine, Pediatrics, Surgery, Gynecology and Obstetrics) and other specialty units [23]. A cross sectional study design was employed to assess drug–drug interactions in elderly patients. For this study, individuals aged 60 and above were included based on the UN’s definition of elderly [1] and this is the accepted cutoff for elderly population in Ethiopia [2]. Participants’ medical record was reviewed every 2 days from date of admission to date of discharge to see any added or discontinued treatments. Prescribed drugs being taken concurrently for at least 24 h were included and checked for drug–drug interaction using Micromedex® 2.0 online drug reference [24]. Micromedex® is medical software which gives evidence-based drug information about DDIs and potential ADRs. It categorizes drug combinations into contraindicated, major, moderate, and minor level of interaction based on the mortality and morbidity probabilities on patients.

### Definitions

*Concurrent or concomitant drugs* Is defined as the concurrent use of drugs as prescribed by one or more different medical doctors not necessarily on the same day [25].

*Comorbidity* Is the presence of more than one distinct medical condition in an individual [26].

*Contraindicated DDI* The drug-pair is contraindicated for concurrent use [24].

*Major DDI* The interaction may have risk of death and/or require medical intervention to minimize or prevent some serious negative outcomes [24].

*Moderate DDI* It may have harmful effect on patient’s condition and can require change in the prescription [24].

*Polypharmacy* Is defined as the use of many drugs at the same time or the administration of an excessive number of drugs [27]. There is no standard cut point with regard to the number of medications that is agreed upon for the definition of polypharmacy. In this study, polypharmacy was operationalized to mean five drugs or more.

A sample of 140 elderly patients was studied. The sample size was determined using the formula for estimation of single proportion [n = (Z·α/2)^2^ p(1 − p)/d^2^] where Z = standard normal variable at 95 % confidence level (1.96), p = the prevalence of potential drug–drug interaction assumed to be 50 % and finally adjusted using correction formula for finite population. Study participants were selected by systematic random sampling techniques. List of admitted elderly patient at a particular time was considered as sampling frame. Sampling interval was obtained by dividing the number of elderly patients likely to be admitted during the study period divided by the sample size which gives 2 (300/140 = 2). The first patient was selected randomly and every other patient was included in the study.

Data were collected using a structured and pretested format that contains patient identification, medical profile and drugs prescribed. The data collectors were six final year pharmacy students, trained on how to approach study participants, ask for informed consent, abstract data from patient records and maintain the confidentiality of the collected data. Prior to the actual data collection, a pretest was done on six elderly patients (not included in this study) to check the practicability of the data collection format and procedures and to assess the performance of data collectors.

The evaluation of drug–drug interaction and data analysis was done by the investigators. Micromedex® 2.0 online drug reference [24] was used to check and describe the types of drug–drug interaction. Data were analyzed using statistical package for social sciences; for windows version 20 statistical software. The data were first checked for completeness then edited, cleaned and entered into the software. Descriptive statistics (frequencies, percentage, mean and standard deviation) were used to present counts, proportions and averages. Binary logistic regression model was used to analyze factors associated with occurrence of drug interaction. Output of the logistic regression was expressed as adjusted odds ratios at 95 % confidence intervals. P value of <0.05 was considered statistically significant.

## Results

The medical records of 140 patients aged 60 years and above were utilized for the study. The mean age (±SD) of participants was 68 (±7) years. Almost half (52.1 %) of the study participants were male. The mean hospital stay of patients was 13 (±9) days. 38 % of the participants had comorbid conditions while 17.8 of them had a single diagnosis. Majority (61.4 %) of the patients had a diagnosis of cardiovascular (CV) and/or renal disease. The common CV diseases were heart failure (22.8 %), followed by hypertension (20 %) while 6.4 % of patients had both medical conditions. Infectious diseases were diagnosed in 58.6 % of patients. Pneumonia accompanied for 32 %, followed by tuberculosis (15 %) and urinary tract infection (5.7 %). The third ranked class of diagnosis was hematologic and thromboembolic disorders.

A total of 814 drugs were prescribed during the mean hospital stay of 13 (±9) days, with an mean of 6 (±4) medications per patient. Fifty-nine (42.2 %) patients had <5 prescribed drugs while 40 and 17.8 % of them had 5–8 and more than 8 prescribed drugs, respectively. Ceftriaxone was the most commonly used medication, prescribed in 55.75 % of the participants.

According to Micromedex® 2.0 online drug reference, nearly two-third (62.2 %) of the participants were exposed to at least one potential DDI. Among those 3.6, 32.9 and 25.7 % of elderly patients were prescribed a contraindicated drug combination, at least one major and at least one moderate DDI respectively. Clarithromycin with either simvastatin or ciprofloxacin was the only contraindicated drug combination in this study (Table 1).

**Table 1:** List of contraindicated drug combinations and potential risk

Drug combinations	Number of patients	Documentation^a^	Potential risks
Clarithromycin simvastatin	3	Good	Increased risk of myopathy or rhabdomyolysis
Clarithromycin ciprofloxacin	1	Fair	Increased risk of QT interval prolongation
Total	4		

^a^ Documentation based on available studies or reports: good, studies strongly suggest that the interaction exists except proof of well-controlled studies; fair, available evidences are poor, but the interaction is suspected on the basis of pharmacologic considerations; or, evidences are good for an interaction of pharmacologically similar drug [24]

The most frequently prescribed major DDIs were the combination of aspirin with either heparin or clopidogrel; warfarin with quinolone and macrolide antibiotics; and spironolactone with enalapril (Table 2).

**Table 2:** List of major drug–drug interactions with their potential risks

Interacting drugs	Number of patients	Documentation^a^	Potential risks
Anti TB(HRZ) + phenytoin	2	Good	Decreased phenytoin and/or rifampin exposure
Aspirin + cimetidine	1	ND	
Aspirin + clopidogrel	5	Fair	Increased risk of bleeding
Aspirin + enoxaparin	2	Good	Increased risk of bleeding
Aspirin + fluoxetine	1	Good	Increased risk of bleeding
Aspirin + omeprazole	1	ND	
Ciprofloxacin + amitriptyline	1	Fair	Increased risk of QT interval prolongation
Ciprofloxacin + doxorubicin	1	Fair	Increased doxorubicin exposure
Clarithromycin + amitriptyline	1	Fair	Increased risk of QT-interval prolongation
Clarithromycin + amlodipine	1	Excellent	Increased amlodipine exposure
Clarithromycin + digoxin	2	Excellent	Digoxin toxicity (nausea, vomiting, arrhythmias)
Clarithromycin + nifedipine	1	Moderate fair	Increased nifedipine plasma concentrations
Clarithromycin + tramadol	3	Fair	Increased risk for seizures, serotonin syndrome, and opioid-related toxicity
Clarithromycin + atorvastatin	1	Good	Increased risk of myopathy or rhabdomyolysis
Clopidogrel + cimetidine	3	Fair	Reduction in clinical efficacy of clopidogrel
Clopidogrel + diclofenac	1	Excellent	An increased risk of bleeding
Clopidogrel + omeprazole	1	Excellent	Reduction in clinical efficacy of clopidogrel and increased risk for thrombosis
Cyclophosphamide + allopurinol	3	Good	Cyclophosphamide toxicity (bone marrow suppression, nausea, vomiting)
Cyclophosphamide + doxorubicin	3	Fair	Increased risk of cardiomyopathy
Heparin + aspirin	14	Fair	Increased risk of bleeding
Heparin + enoxaparin	1	Fair	Increased risk of bleeding
Heparin + clopidogrel	4	Fair	Increased risk of bleeding
Heparin + diclofenac	1	Fair	Increased risk of gastrointestinal bleeding
Heparin + indomethacin	3	Excellent	Increased risk of gastrointestinal bleeding; reduced effectiveness of indomethacin for the treatment of patent ductus arteriosus
Hydralazine + metformin	1	ND	
Hydralazine + norfloxacin	1	ND	
Insulin + norfloxacin	2	Fair	Changes in blood glucose and increased risk of hypoglycemia or hyperglycemia
Insulin + ciprofloxacin	2	Fair	Changes in blood glucose and increased risk of hypoglycemia or hyperglycemia
Potassium chloride + enalapril	2	Good	Hyperkalemia
Simvastatin + azithromycin	2	Good	An increased risk of rhabdomyolysis
Simvastatin + ciprofloxacin	2	Good	Increased risk of myopathy or rhabdomyolysis
Spironolactone + digoxin	2	Good	Increased digoxin exposure
Spironolactone + enalapril	10	Good	Hyperkalemia
Spironolactone + potassium chloride	4	Fair	Hyperkalemia
Tramadol + chlorpromazine	2	Fair	Increased risk of seizures
Tramadol + amitriptyline	1	Fair	Increased risk of seizures, serotonin syndrome (hypertension, hyperthermia, myoclonus, mental status changes), opioid toxicity, and increased concentrations of tramadol and decreased concentrations of tramadol active metabolite
Warfarin + aspirin	3	Fair	Increased risk of bleeding
Warfarin + azithromycin	2	Good	Increased risk of bleeding
Warfarin + ciprofloxacin	3	Good	Increased risk of bleeding
Warfarin + clarithromycin	3	Good	Increased risk of bleeding
Warfarin + metronidazole	6	Good	Increased risk of bleeding
Total	105		

^a^ Documentation based on available studies or reports: excellent, the interaction has been clearly demonstrated in well-controlled studies; good, studies strongly suggest that the interaction exists except proof of well-controlled studies; fair, available evidences are poor, but the interaction is suspected on the basis of pharmacologic considerations; or, evidences are good for an interaction of pharmacologically similar drug [24]

*ND* not documented, *Anti TB (HRZ)* anti tuberculosis (isoniazid/rifampicin/pyrazinamide)

There was no significant difference in the occurrence of DDI with respect to age, sex, presence of co-morbidities and length of hospital stay. DDI was found significantly associated with increase in number of drugs (polypharmacy) (P = 0.00; adjusted odds ratio 4.047; 95 % confidence interval 1.867–8.775) (Table 3).

**Table 3:** Statistical association of variables with drug–drug interaction

Variable	Category	DDI		P value	Adjusted odds ratio
		No	Yes		
Sex	Male	28	45		
	Female	25	42	0.531	0.782 (0.363–1.686)
	Total	53	87		
Age	60–69	26	50		
	70–79	18	30	0.858	0.927 (0.406–2.12)
	>80	9	7	0.143	0.392 (0.112–1.373)
	Total	53	87		
Comorbidities	No	15	10		
	Yes	38	77	0.241	0.557 (0.209–1.483)
	Total	53	87		
Hospital stay (days)	<10	35	38		
	≥10	18	49	0.168	0.168 (0.795–3.726)
	Total	53	87		
Number of drugs (polypharmacy)	<5	35	24		
	≥5	18	63	0.000^a^	4.047 (1.867–8.775)
	Total	53	87		

*DDI* drug–drug interaction

^a^ Statistically significant association

## Discussion

The mean (±SD) number of drugs prescribed per patient in this study was 6 (±4) which is almost similar to a study in Taiwan [28] 5.8 ± 2.4 but lower than study reports from Puducherry [29], India [30]; which was 7.61 ± 3.37 and 9.15 ± 0.03, respectively. These differences could be due to the difference in study set-up, health insurance policy, and difference in burden of co-morbidity and medication use pattern in these settings. In the current study, there is similar burden of infectious diseases (58.6 %) and cardiovascular diseases (61.4 %), where as in developed countries non-communicable diseases including cardiovascular diseases are more common than infectious diseases. Unlike in developed countries, health insurance policy for the elderly which provides more access to medications is not introduced in this study set-up. In this study, 62.2 % of the respondents were exposed to at least one potential DDI [including contraindicated (3.6 %), major (32.9 %) and moderate (25.7 %)]. The prevalence of major and moderate DDI in the current study is in line with the findings by Luca et al. [31] and Lea et al. [32], which reported a prevalence of 63.5, 60.5 % potential DDIs respectively. In contrast to this study, lower prevalence of DDIs was reported from other studies focusing on elderly outpatients [33–35]. This indicates that drug interactions are more common in inpatients set-up as compared to outpatients probably due to the difference in number of drugs prescribed per patient.

In this study polypharmacy (taking five drugs or more) was identified as predictor for the occurrence of DDI (P = 0.00; AOR 4.047; 95 % CI 1.867–8.775). A number of other studies have also identified an increase number of medications a predictor of DDI in the elderly [6, 9, 15, 30, 34–41]. A study from Brazil reported that the potential drug interaction risk when patients are taking 2–3, 4–5 and 6–7 medications was 39, 88.8 and 100 %, respectively [36]. Nearly 50 % of elderly patients take one or more medications that are not medically necessary [37]. However, in the context of the elderly, polypharmacy does not necessarily mean inappropriate. A number of elderly patients may be prescribed combination of drugs to achieve synergic therapeutic response. As the use of many drugs in the elderly is an avoidable due to the comorbidities they have, it is important not to discard important medications because of the potential risk of drug interactions. Many of the drug interactions can be minimized by using alternative medications but those that are not require awareness of the interaction to allow for proper management and appropriate dose adjustment. In reality however we must be knowledgeable not only about DDIs; indeed, a broad understanding of how to use various drugs safely in our patients is essential.

The DDI in the current study are theoretical or potential DDIs. Though the incidence of actual DDIs is lower than that of potential DDIs, some studies have found as high as 25–47 % clinically relevance potential DDIs in the elderly [33, 41]. The occurrence of clinically important interactions depends in the presence of specific risk factors such as polypharmacy, comorbidity, age, therapeutic range and dosage of the drug [42]. A research has clearly established a strong relationship between polypharmacy leading to DDI and negative clinical consequences [14, 37]. Thus, the current study is important to make awareness on the dangerous potential interactions that could have negative clinical consequences and identify risky groups.

In this study a contraindicated drug combination was observed in four (3.6 %) patients. These interactions were involving clarithromycin with either simvastatin or ciprofloxacin. In a panel of experts, these interactions were among the highly clinically significant drug–drug interactions identified as contraindicated for concurrent use [43]. The interaction between clarithromycin and simvastatin increases the risk of myopathy or rhabdomyolysis [24, 43] and myopathy was the third commonest ADR caused by DDI next to gastrointestinal bleeding and hyperkalemia [15]. Concurrent use of clarithromycin and ciprofloxacin is contraindicated due to the increased risk of QT interval prolongations [24, 43]. Probably these interactions can be avoided with the use of alternative antibiotics with similar spectrum of activity but less potential for interactions.

The most frequently prescribed major DDIs in the current study were the combination of aspirin with anticoagulants (heparin or warfarin) and/or clopidogrel; warfarin with antibiotics and spironolactone with enalapril in decreasing order of prevalence. Low-dose aspirin with clopidogrel and/or anticoagulants is increasingly prescribed in combinations to the elderly to prevent atherothrombotic events. A number of studies have identified these interactions responsible for the greatest number of serious bleedings [15, 30, 39, 44]. However, these combinations are sometimes unavoidable and might be indicated. Thus, careful monitoring and evaluation of the risk of actual drug-interaction and benefits of continuing both medications is vital. When co-administration of these drugs is required laboratory values such as international normalized ratio, signs and symptoms of bleeding should be closely monitored.

Co-administration of potassium sparing diuretic (spironolactone) with potassium supplements or angiotensin converting enzyme (ACE) inhibitors like enalapril where observed in this study. Concomitant use of these agents increases the risk of hyperkalemia and the elderly are more sensitive to this adverse effect. Other studies also reported these combinations as one of the clinically relevant DDI [15, 44–47]. A nested case control study by Juurlink et al. [47], showed that elderly patients treated with ACE inhibitor admitted with hyperkalemia were about 20 times more likely to have been treated with potassium sparing diuretic (adjusted odds ratio 20.3; 95 % confidence interval 13.4–30.7).

Many of the potential DDI in the elderly can be avoided with close patient monitoring or the use of alternative medications. However, it may be difficult for clinicians to memorize the thousands of DDIs and their clinical significance. Clinical pharmacist can play a role in identification and monitoring of potential DDIs hence to make appropriate dosage or therapy adjustments. Studies are conflicting whether clinical pharmacist interventions such as pharmaceutical care and educational campaigns in improving distal health outcomes [37, 48, 49]. However, overall improvement in quality of prescribing, use of appropriate polypharmacy hence reduced potential DDIs were observed [37, 49]. Indeed, decision support systems and information technologies are nowadays increasingly utilized to prevent severe DDIs. Despite the alert fatigue that has been identified as a major limitation in using these technologies, clinical pharmacist assisted computerized decision support systems was found to be efficient and offer an opportunity to detect potential DDIs [50].

The current study provides insight into the prevalence of potential DDIs in elderly inpatients in a resource constrained setting. In addition, it was possible to identify polypharmacy as a risk factor for the occurrence of DDI in the elderly which have been observed in other studies. However, the study has some limitations: one the study was done on small number of elderly patients compared to other studies. Second, being a cross sectional study which was carried out at one time point, it was not possible to see the outcome of the potential DDI or the actual occurrence of the interactions from a clinical viewpoint. Further longitudinal studies using larger number of participants are necessary.

## Conclusion

The finding of present study reveals that nearly two-third of the elderly patients are exposed to at least one potential DDIs. Elderly patients on five or more medications needs close monitoring as they are four times at higher risk of having DDIs (Additional file 1). Identifying and preventing potentially harmful DDIs is a critical component of a pharmacist’s mission and the clinical pharmacist must remain vigilant in monitoring potential DDIs and making appropriate dosage or therapy adjustments.

## Additional files

10.1186/s13104-016-2238-5 Dataset.
10.1186/s13104-016-2238-5 STROBE Statement—checklist of items that should be included in reports of observational studies.

## Abbreviations

ADRs: adverse drug reactions
AOR: adjusted odds ratio
ACE: angiotensin converting enzyme
ARH: Ayder Referral Hospital
CI: confidence interval
CV: cardiovascular
DDIs: drug–drug interactions
ND: not documented
SD: standard deviation
SPSS: statistical package for social sciences
UN: United Nations
