# Supplementary material for: Potential drug–drug interactions among elderly patients admitted to medical ward of Ayder Referral Hospital, Northern Ethiopia: a cross sectional study
Source: BMC Res Notes. 2016 Sep 1;9(1):431. doi: 10.1186/s13104-016-2238-5 (PMC5009535; doi:10.1186/s13104-016-2238-5)
Supplement: Supplementary file 2 — 10.1186/s13104-016-2238-5 STROBE Statement—checklist of items that should be included in reports of observational studies. [file 13104_2016_2238_MOESM2_ESM.doc]

STROBE Statement—checklist of items that should be included in reports of observational studies

|  | | | Item No | Recommendation |
| --- | --- | --- | --- | --- |
| Title and abstract | | | 1 | (a) Potential drug-drug interactions among elderly patients admitted to medical ward of Ayder Referral Hospital, Northern Ethiopia: a cross sectional study |
|  |  |  |  | (b) Despite the fact that the elderly are at high risk of having drug interaction and potential adverse outcomes, studies in this regard are scarce in resource limited settings like Ethiopia. The aim of this study was to assess the prevalence and determinants of potential drug-drug interaction in elderly patients admitted to medical ward of Ayder referral hospital in Northern Ethiopia. A cross sectional study was conducted among elderly inpatients aged 60 years and above. The study was conducted from February to May, 2014. One hundred forty patients were participated in the study. The mean age (±standard diversion) of participants was 68 (±7) years. A total of 814 drugs were prescribed with an average of 6 (± 4) medications per patient during a 13 (± 9) days of hospital stay. About two third (62.2%) of the respondents were exposed to at least one potential drug-drug interaction. Among these 3.6%, 32.9% and 25.7% of patients had taken contraindicated drug combination, at least one major and at least one moderate drug-drug interaction, respectively. Patients with five or more prescribed medications were four times at risk of having drug- drug interaction (p-value=0.00; adjusted odds ratio=4.047; 95% confidence interval=1.867- 8.775). Drug-drug interaction in elderly patients was common in this resource limited set-up. Awareness creation and clinical pharmacist interventions aimed at reducing exposure and minimizing the risk associated with potentially harmful drug combinations are needed. |
| Introduction | | | | |
| Background/rationale | | | 2 | The International Conference on Harmonization considers older people a ‘special population’, as they differ from younger adults in terms of comorbidity, polypharmacy, pharmacokinetics and greater vulnerability to adverse drug reactions (ADRs). Drug-drug interactions (DDI) are more likely to happen in the elderly because they tend to use multiple medications and have altered pharmacokinetics. Drug interactions are an important cause of ADRs and hospital admission. Despite the fact that the elderly patients are at high risk of having drug interaction and potential adverse outcomes, studies in this regard are scarce in resource limited settings like Ethiopia. |
| Objectives | | | 3 | The specific objectives of this study were to assess the prevalence and types of potential DDIs; to identify the determinants of potential DDIs among elderly patients aged 60 years and above admitted to medical ward of Ayder referral hospital in Northern Ethiopia. |
| Methods | | | | |
| Study design | | | 4 | A cross sectional study design was employed to assess drug-drug interactions in elderly patients. The study was conducted by reviewing prescribed medications in the patient medical record. Prescribed drugs being taken concurrently for at least 24 hours were included and checked for drug-drug interaction using Micromedex® 2.0 online drug reference. |
| Setting | | | 5 | The study was conducted in Ayder Referral Hospital (ARH) located in Mekelle city of Tigray Regional State, northern Ethiopia. ARH is a teaching and the highest level medical facility in the Northern part of the country. It provides general inpatient and outpatient medical services to its catchment population. Data was collected from February to May 2014. |
| Participants | | | 6 | The source population was all elderly patients admitted to medical ward of Ayder referral hospital. Individuals aged 60 and above based on the UN’s definition of elderly and willingness to participate based on the consent obtained from the patient himself or his/her care giver were the eligibility criteria to include in this study. Participants were selected by systematic random sampling techniques. The first patient was selected randomly and every other bed of elderly patient was included in the study. |
|  |  |  |  |  |
| Variables | | | 7 | Dependent variable: drug-drug interaction  Independent variables: Age, sex, number of co-morbidities, length of hospital stay and number of drugs (polypharmacy). |
| Data sources/ measurement | | | 8 | Presence of potential DDI was determined/checked using Micromedex® 2.0 online drug reference. This software classifies potential DDI as contraindicated, major and moderate level of interaction based on the mortality and morbidity evidences seen in patients.  Age, sex, number of co-morbidities, length of hospital stay and number of drugs prescribed were obtained from the patient medical record. Age was classified as 60 to 69; 70 to 79; 80 and above which is a common classification of elderly in studies done in developing countries. Length of hospital stay was classified as less than ten or ten and above based on the mean hospital stay. Number of co-morbidities was classified as no (for participant without co-morbidity) versus yes (for participants with one and above co-morbidities). There is no standard cut point with regard to the number of medications that is agreed upon for the definition of polypharmacy. In this study, polypharmacy was operationalized to mean five drugs or more. |
| Bias | | | 9 | A systematic sampling had been employed to minimize selection bias. As the data was collected from patient medical records, there was no any information biases introduced into our study. In addition data was collected by trained individuals other than the investigators. |
| Study size | | | 10 | The sample size was determined using the formula for estimation of single proportion [n = (Z α/2)^2^ p(1-p)/d^2^] where Z=standard normal variable at 95% confidence level (1.96), p= the prevalence of potential drug-drug interaction assumed to be 50% and finally adjusted using correction formula for finite population. Finally, a sample of 140 elderly patients was studied. |
| Quantitative variables | | | 11 | The quantitative variables were entered and analysed using SPSS for windows version 20 statistical software. Number, percentage, mean and standard deviation were used to present counts, proportions and averages of these variables. |
| Statistical methods | | | 12 | (a) Binary logistic regression model was used to identify factors associated with occurrence of drug-drug interaction. |
|  |  |  |  | (b) No subgroups |
|  |  |  |  | (c) All collected data were first checked for completeness, and any filled tools with missing data were not included for analysis. |
|  |  |  |  | (e) There were no any significant uncertainties in the output of our data. However we had difficulties in deciding some of the variables. Defining groups of older people was difficult in this study and it can have lower limits of 60, 65, 70 or 75 years, or any value in between. The age 65 and above, roughly equivalent to retirement ages in most developed countries, is used to define the elderlly. However, this is not adapted well to the situation in developing countries like Africa. The United Nation (UN) agreed cutoff is 60 and above to refer to the older population and this definition has gained acceptance in Ethiopian context as it coincides with the country's official retirement age. Finally the age 60 was used to define elderly which was in line with other studies done in developing countries. Unlike polypharmacy, there was no statistical association between comorbidity and occurrence of drug interaction. As comorbidity increases so do polypharmacy and it is expected both variables to conside with the outcome of interest. This uncertainty could be introduced due to the differences in measurement of each variable. Co-morbidity was considered in the presence of more than one distinct medical condition in an individual. There is no standard cut point with regard to the number of medications that is agreed upon for the definition of polypharmacy. In this study, polypharmacy was operationalized to mean five drugs or more. Individuals with five drugs or more are more likely to have two or more co-morbidities. |
| Results | | | | |
| Participants | 13 | (a) 451 patients admitted to the internal medicine ward during the study period were examined for eligibility. 140 patients which were aged 60 and above and willing to participate were included in the study. Concurrent medications being taken together for at least 24 hours were included for analysis. | | |
|  |  | (b) All eligible participants identified were willing to participate in the study | | |
| Descriptive data | 14 | (a) The mean age (±SD) of participants was 68 (±7) years. Almost half (52.1%) of the study participants were male. The mean hospital stay of patients was 13 (± 9) days. Thirty eight percent of the partcipants had comorbid conditions while 17.8 of them had a single diagnosis. Majority (61.4 %) of the patients had a diagnosis of cardiovascular (CV) and/or renal disease. | | |
|  |  | (b) No participant with missing data | | |
| Outcome data | 15 | A total of 814 drugs were prescribed during the mean hospital stay of 13 (± 9) days, with an average of 6 (± 4) medications per patient. Nearly two third (62.2%) of the participants were exposed to at least one potential DDI. Among those 3.6%, 32.9% and 25.7% of elderly patients were prescribed a contraindicated drug combination, at least one major and at least one moderate DDI respectively.—Report numbers of outcome events or summary measures | | |
| Main results | 16 | (a) Adjusting for other variables such as age, sex, presence of co-morbidities and length of hospital stay; drug drug interaction was found significantly associated with the use of five or more drugs (polypharmacy) (p-value=0.00; adjusted odds ratio=4.047; 95% confidence interval=1.867- 8.775). | | |
|  |  | (b) Age and length of hospital stay were the two continuous variables that had been categorized. We specified age to mean in years and length of hospital stay in days. Age was classified as with the following boundaries: 60 to 69; 70 to 79; 80 and above. Length of hospital stay was classified as a bench mark of greater than or less than 10. | | |
| Other analyses | 17 | No subgroups analyses or other analysis done | | |
| Discussion | | | | |
| Key results | 18 | Nearly two third (62.2%) of the respondents were exposed to at least one potential DDI (3.6% contraindicated, 32.9% major and 25.7% moderate level of interaction) and the first objective on the prevalence and types of potential DDIs has meet. polypharmacy (taking five drugs or more) was identified as predictor for the occurrence of drug-drug interaction in the elderly. This also meats the second objective which was to identify the determinants of potential DDIs. | | |
| Limitations | 19 | This study has some limitations. One, the study was done on small number of elderly patients compared to other studies. Second, being a cross sectional study which was carried out at one time point, it was not possible to see the outcome of the potential DDI or the actual occurrence of the interactions from a clinical viewpoint. Further longitudinal studies using larger number of participants are necessary.  There was no potential bias introduced in this study | | |
| Interpretation | 20 | Drug-drug interactions are also common in the elderly patients treated in resource limited set-ups. Elderly patients taking a number of medications needs close monitoring as they at higher risk of having drug-drug interactions. Awareness creation to prescribers and clinical pharmacist interventions to identify and prevent potentially harmful DDIs is crucial. | | |
| Generalisability | 21 | The current study provides insight into the prevalence of potential DDIs in elderly inpatients in a resource constrained setting. In addition, it was possible to identify polypharmacy as a risk factor for the occurrence of DDI in the Elderly which have been also observed in other studies. The study finding might be generalisable to elderly patients being treated in other resource constrained settings. As the study is the first in its kind in Ethiopian set-up, it can be repeated in other areas with inclusion of the study limitations. | | |
| Other information | | | | |
| Funding | 22 | We obtained no funding for this study | | |

Note: An Explanation and Elaboration article discusses each checklist item and gives methodological background and published examples of transparent reporting. The STROBE checklist is best used in conjunction with this article (freely available on the Web sites of PLoS Medicine at http://www.plosmedicine.org/, Annals of Internal Medicine at http://www.annals.org/, and Epidemiology at http://www.epidem.com/). Information on the STROBE Initiative is available at www.strobe-statement.org.
